# Simultaneous isolation of *Chryseobacterium gleum* from bloodstream and respiratory tract: first case report from India

**DOI:** 10.1099/jmmcr.0.005122

**Published:** 2017-10-16

**Authors:** Vidhi Jain, Nayani Amrin Fatema Afzal Hussain, Tasneem Siddiqui, Chinmoy Sahu, Malay Ghar, Kashi Nath Prasad

**Affiliations:** Department of Microbiology, Sanjay Gandhi Post Graduate Institute of Medical Sciences, Lucknow, India

**Keywords:** *Chryseobacterium gleum*, bacteremia, pneumonia, fever, levofloxacin, bacteremia, respiratory distress

## Abstract

**Introduction.** Species of the genus *Chryseobacterium* are emerging healthcare-associated pathogens, often colonizing the hospital environment. There are no clear guidelines available for antimicrobial susceptibility of this organism. In this report we present the first case, to our knowledge, of simultaneous central-line-associated bloodstream infection (CLABSI) and ventilator-associated pneumonia (VAP) due to *Chryseobacterium gleum* from India.

**Case presentation.** A 62 years old man with a history of a road traffic accident 1 month previously was referred to our center for further management. He developed features of sepsis and aspiration pneumonia on day 3 of admission. Four blood cultures (two each from central and peripheral lines) and two tracheal aspirate cultures grew pure yellow colonies of bacteria. Both matrix assisted laser desorption ionization time of flight mass spectrometry, (MALDI-TOF MS; bioMérieux, Marcy-L'Etoile, France,) and BD Phoenix (BD Biosciences, Maryland, USA) identified the organism as *C. gleum*. However, BD Phoenix failed to provide MIC breakpoints. The isolates of *C. gleum* both from blood and tracheal aspirate showed identical susceptibility patterns: resistant to cephalosporins and carbapenems and susceptible to ciprofloxacin, levofloxacin, amikacin, trimethoprim+sulfamethoxazole, piperacillin–tazobactam, cefoperazone–sulbactam, doxycycline, minocycline and vancomycin. Following levofloxacin therapy, the fever responded within 48 h and procalcitonin levels decreased without removal of the central line or endotracheal tube. However, the patient developed sudden cardiac arrest on day 10 of treatment and could not be resuscitated.

**Conclusion.** Rapid and accurate identification of *C. gleum* in the laboratory, preferably based on MALDI-TOF, is essential for guiding therapy. *C. gleum* responds well to fluoroquinolones without the need to remove indwelling catheters.

## Abbreviations

CLABSI, central-line-associated bloodstream infection; MALDI-TOF MS, matrix assisted laser desorption Ionization time of flight mass spectrometry; NFGNB, Non-fermentative Gram-negative Bacilli; VAP, ventilator-associated pneumonia.

## Introduction

Healthcare-associated infections are a menace for patients and clinicians alike. Non-fermentative Gram-negative bacilli (NFGNB) are commonly found on moist hospital surfaces, like washbasins and dressing trolleys [1]. If hand hygiene is inadequate, they can be passed on to patients during contact with healthcare workers [2]. They can colonize mechanical devices, causing device-associated infections like central-line-associated bloodstream infection (CLABSI) and ventilator-associated pneumonia (VAP) [1, 2]. These infections usually respond to broad-spectrum antibiotics like carbapenem and colistin.

The Flavobacterium group contains peculiar NFGNB, which are resistant to broad-spectrum antibiotics like carbapenems and colistin. Instead, they respond to antibiotics like fluoroquinolones and macrolides. The Flavobacterium IIb group producing yellow pigment has been deemed to be distinct from other flavobacteria, owing to differences in 16S rRNA and DNA–DNA homology, by Holmes *et al.* in 1984 [3]. They were renamed as species of the genus *Chryseobacterium* and proposed as a separate genus on the basis of rRNA cistron studies by Vandamme *et al*. in 1994 [4] *Chryseobacterium indologenes* has been noted as an emerging pathogen associated with CLABSI and VAP [5]. *Chryseobacterium gleum* is an infrequently isolated member of the genus *Chryseobacterium* worldwide and has been implicated in urinary tract infection, pneumonia and pyonephrosis in India [6–8]. We present the first case, to our knowledge, of CLABSI and VAP due to *Chryseobacterium gleum* from India.

## Case report

A 62 years old man with a history of a tentorial bleed and fractures of the left clavicle and left ulna, after a road traffic accident 1 month ago, was referred to our center for further management. The patient was a known hypertensive and diabetic for the last 10 years, with chronic renal disease since 2015 and had undergone coronary artery bypass grafting (CABG) for coronary artery disease in 2009, after which he had been put on aspirin. He was a non-smoker, non-alcoholic, non-vegetarian and a clerk by occupation.

He was initially admitted to a private nursing home, where he started developing labored breathing and altered sensorium after two weeks, with five to six seizures per day and two episodes of vomiting. He continued to deteriorate slowly over the next two weeks with insidious onset low-grade fever and serum creatinine rising to 2 mg dl^−1^ (176.84 µmol l^−1^).

At the time of presentation to our hospital in October 2016, the patient was in altered sensorium with a Glasgow coma scale E2V2M4. He had mild pallor, no icterus, no clubbing, no pedal oedema and jugular venous pressure was not raised. His pulse rate was 100 min^−1^, blood pressure 100/60 mm Hg. Respiratory system examination revealed few rhonchi at the base of right lung. Neurosurgery consultation was taken and a conservative approach was adopted in view of hyponatremia, altered sensorium and poor prognosis. He was admitted to the ICU and put on mechanical ventilation with noradrenaline support and chest physiotherapy.

He developed a fever spike on day 3 of admission and blood cultures from both central and peripheral lines collected on the consecutive first three days of fever were sent to the laboratory. Complete blood count showed leukocytosis (14 800 mm^−3^) with raised neutrophils (86 %) and reduced lymphocytes (20 %). The patient’s serum creatinine level was 3.55 mg dl^−1^ (313.89 µmol l^−1^). His blood urea nitrogen was 79 mg dl^−1^ (28.21 mmol l^−1^). Serum procalcitonin was raised (2.35 ng ml^−1^). He was administered imipenem (500 mg QID) and teicoplanin (400 mg OD). His general condition continued to deteriorate. On the next day his chest X-ray showed patchy shadows in the lower respiratory tract, suggestive of aspiration pneumonia. Tracheal aspirates were also sent for culture on three consecutive days. Colistin (three million units) was added empirically.

**Fig. 1.**
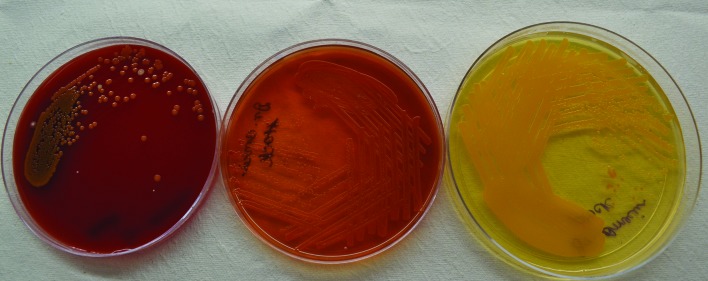
Yellow-pigmented colonies of *Chryseobacterium gleum* on Mueller–Hinton agar that change colour to red instantly after the addition of 10 % KOH, useful for provisional identification of species of the genus *Chryseobacterium.*

Four blood cultures (two each from central and peripheral lines) and two tracheal aspirate cultures grew pure yellow colonies on 5 % sheep blood agar and MacConkey agar. The pigment was non-diffusible when grown in nutrient broth. Upon subculture, the yellow colonies grew on overnight incubation at 37 °C on blood agar and they were yellow opaque, low convex, glistening colonies with a non-diffusible pigment. They also grew on MacConkey agar within 48 h of incubation at 37 °C. The organism was provisionally identified as a Gram-negative, non-motile, catalase- and oxidase-positive bacillus. When plated on Mueller–Hinton agar, the yellow colonies immediately turned red on addition of 10 % KOH, indicating the presence of a flexirubin type of pigment (Fig. 1). Matrix assisted laser desorption Ionization time of flight mass spectrometry (MALDI-TOF MS; BioMérieux, Marcy-L'Etoile, France) identified the organism as *C. gleum*. BD Phoenix (BD Bioesciences, Maryland, USA) also identified the organism as *C. gleum*, however, it failed to provide MIC breakpoints for the organism despite repeated testing.

Antimicrobial susceptibility testing was performed by the Kirby–Bauer disc diffusion method as per clinical and laboratory standards institute (CLSI) protocols for other non-Enterobacteriaceae, non-fermenters other than *Pseudomonas aeruginosa* and members of the genera *Stenotrophomoas* and *Burkholderia* [9]. All the six clinical isolates of *C. gleum* showed identical susceptibility patterns: resistant to amoxycillin (10 µg), cefotaxime (30 µg), ceftazidime (30 µg), cefoperazone (75 µg), cefepime (30 µg), imipenem (10 µg), meropenem (10 µg), gentamicin (10 µg), tobramycin (10 µg), clindamycin (15 µg) and erythromycin (15 µg). They were susceptible to ciprofloxacin (5 µg), levofloxacin (5 µg), amikacin (30 µg), trimethoprim+sulfamethoxazole (15 µg), piperacillin–tazobactam (100/10 µg), cefoperazone–sulbactam (75/10 µg), doxycycline (30 µg), minocycline (30 µg) and vancomycin (30 µg). An E-test was performed for some selected antibiotics. The organism was resistant to imipenem (>32 µg ml^−1^), meropenem (>32 µg ml^−1^), doripenem (>32 µg ml^−1^) and colistin (>256 µg ml^−1^). The MIC for vancomycin was 3 µg ml^−1^.

The patient developed generalized seizures, ventilator dyssynchrony and severe acute respiratory distress syndrome. He did not improve despite colistin, imipenem and teicoplanin therapy. The blood culture report with final identification and antimicrobial susceptibility profile was available on day 4, when the treating clinician added levofloxacin 750 mg IV OD. Following fluoroquinolone therapy, the fever responded within 48 h and procalcitonin levels decreased to 0.8 ng ml^−1^ after 7 days, without removal of the central line or endotracheal tube. Unfortunately, the patient continued to deteriorate neurologically, with frequent seizure activity. He developed sudden cardiac arrest on day 10 of treatment and could not be resuscitated.

## Discussion

The emergence of species of the genus *Chryseobacterium* as medically relevant pathogens was first described by the SENTRY study, which reported isolates of members of the genus *Chryseobacterium* to constitute 0.27 % of NFGNB obtained from clinical specimens across 16 countries [10]. Out of 50 strains of members of the genus *Chryseobacterium* documented by the authors, *Chryseobacterium* *meningosepticum* (now *Elizabethkingia meningoseptica*) was the most common species, while *C. gleum* was the least frequently isolated species, with only two strains isolated over the five-year study period.

Since then, there have been a few case reports of *C. gleum* from clinical specimens like blood, sputum, urine and pus [6, 8, 11–16]. A study conducted in Taiwan revealed that *C. gleum* had the ability to form biofilms [11]. However, its biofilm-forming potential appeared to be much lower than that of *E. meningoseptica*, indicating its lower pathogenic potential [11]. Bloodstream infection due to *C. gleum* has been reported in only two patients in a single study from Taiwan [11]. Interestingly, one of those patients had suffered from head trauma and grew *C. gleum* from a central venous catheter, similar to the patient profile reported by us. However, there is no data available regarding the results of peripheral blood cultures in that patient, so the possibility of colonization cannot be ruled out. The isolation of *C. gleum* from bloodstream infections has not been previously documented from India, to the best of our knowledge.

Though *C. gleum* pneumonia has been reported previously from our country [7], this is the first report, to our knowledge, of simultaneous bloodstream infection and pneumonia due to *C. gleum* from India. Such isolation of *C. gleum* from two distinct clinical specimens from the same patient has, to our knowledge, only been reported once previously, from a patient in Croatia [14]. Reports of *C. gleum* isolation from respiratory tract have been summarized in Table 1. The accurate identification of the organism as *C. gleum* was performed using two automated systems based on different principles. The BD Phoenix automated system is based on an array of biochemical tests, while MALDI-TOF MS uses mass spectrometry, which has been demonstrated to have 100 % precision for identification of *C. gleum* [12]. Our strain grew well on MacConkey agar, contrary to some previous reports [7, 17].

**Table 1.** Summary of reports of isolation of *Chryseobacterium gleum* from the respiratory tract to date

	**Author**	**Year**	**Country**	**Patient profile**	**Treatment given**	**Response to therapy**
1	Lambiase *et al.* [17]	2007	Italy	Two patients with cystic fibrosis	na	na
2	Lo and Chang [11]	2014	Taiwan	Three male and one female hospitalized patients	na	na
3	Virok *et al.* [13]	2014	Hungary	Three neonates with early onset infection	Ciprofloxacin	Responded
4	Brkic *et al.* [14]	2015	Croatia	One female patient with severe malnutrition and hepatic lesion	Piperacillin+tazobactam	Responded
5	Abdalhamid *et al.* [16]	2016	Saudi Arabia	One infant with nephrotic syndrome	Levofloxacin	Responded
6	Rawat *et al.* [7]	2017	India	One infant with chronic granulomatous disease	Piperacillin+tazobactam and cotrimoxazole	Responded
7	Present study	2017	India	One male patient with tentorial bleed after road traffic accident	Levofloxacin	Responded

When an infrequently isolated NFGNB is obtained from a clinical specimen, the significance of its isolation has to be pondered and colonization must be differentiated from true infection. Blood specimens that grew *C. gleum* were collected from central and peripheral vascular access lines on two consecutive days, two samples each taken on the first and second day of fever. BACTEC culture of central lines was positive 3 h prior to that of peripheral lines, meeting the CLABSI criteria for differential time to positivity >120 min. Endotracheal aspirates yielding *C. gleum* were collected over the next two days, when chest X-ray showed signs of pneumonia. Repeated isolation of *C. gleum* from six clinical specimens collected on two consecutive days confirms that it was a true pathogen and not a colonizer.

There are no standard guidelines available from CLSI or EUCAST for the antimicrobial susceptibility testing of members of the genus *Chryseobacterium*. Some authors have used *Staphylococcus* breakpoints for MIC interpretation [8], while others have chosen NFGNB cut offs to interpret results [6, 16]. We used disc diffusion and MIC cut offs for NFGNBs as described previously [9] and interpreted the results accordingly [9]. Our strain was largely susceptible to most groups of antibiotics, especially fluoroquinolones and piperacillin+tazobactam, unlike the strains previously reported from India [6, 8]. Previous studies have tested the effects of vancomycin and clindamycin on these bacteria as an identification marker and not for treatment purposes [8, 10].

Prolonged hospitalization, invasive interventions, indwelling catheters and broad-spectrum antibiotics have been reported to be risk factors for the acquisition of *Chryseobacterium* in critically ill patients [18]. Our patient had a history of a prolonged hospital stay including ICU admission, mechanical devices, like a central line port, endotracheal tube and urinary catheter, and had been exposed to broad-spectrum antibiotics like carbapenems and colistin. He responded clinically to fluoroquinolone therapy, without removal of mechanical devices, similar to the outcome reported by many other authors [8, 11, 14, 16, 19].

### Conclusions

Critically ill patients in ICUs, with mechanical devices, receiving broad-spectrum antibiotics are at risk of healthcare-associated infection due to the emerging pathogen *C. gleum*. Since it is inherently resistant to carbapenems and colistin, its rapid and accurate identification in the laboratory, preferably based on MALDI-TOF MS, is essential for guiding therapy. The organism responds well to fluoroquinolone without the need to remove indwelling catheters.
